# Are tai chi and qigong effective in the treatment of TBI? A systematic review protocol

**DOI:** 10.3389/fnagi.2023.1121064

**Published:** 2023-03-06

**Authors:** Nicole Alexandra Laskosky, Patricia Huston, Wai Ching Lam, Charlotte Anderson, Ya Zheng, Linda L. D. Zhong

**Affiliations:** ^1^Department of Family Medicine, Faculty of Medicine, University of Ottawa, Ottawa, ON, Canada; ^2^Institut du Savoir Montfort (Research), University of Ottawa, Ottawa, ON, Canada; ^3^School of Chinese Medicine, Hong Kong Baptist University, Hong Kong, Hong Kong SAR, China; ^4^School of Biological Sciences, Nanyang Technological University, Singapore, Singapore; ^5^Faculty of Medicine, University of Toronto, Toronto, ON, Canada

**Keywords:** traumatic brain injury, concussion, tai chi, qigong, systematic review

## Abstract

**Background:**

Traumatic brain injury (TBI) adversely affects both young and old and is a growing public health issue. A number of recent trends in managing TBI, such as recommending sub-threshold aerobic activity, tailoring multi-modal treatment strategies, and studying the possible role of low-grade inflammation in those with persistent symptoms, all suggest that the physical and cognitive exercise of tai chi/qigong could have benefit.

**Method:**

Designed in accordance with the Preferred Reporting Items for Systematic Reviews and Meta-Analyses (PRISMA) guidelines, the following databases will be searched: MEDLINE, CINAHL, Cochrane Library, Embase, China National Knowledge Infrastructure Database, Wanfang Database, Chinese Scientific Journal Database, and Chinese Biomedical Literature Database. All clinical trials on mild, moderate and/or severe TBI with tai chi and/or qigong as the treatment group and any comparison group, in any setting will be included. Four reviewers will independently select studies; two reviewers for the English and two for the Chinese databases. Cochrane-based risk of bias assessments will be conducted on all included studies. An analysis will then be conducted with the grading of recommendation, assessment, development, and evaluation (GRADE) instrument.

**Results:**

This review will summarize the clinical trial evidence on tai chi/qigong for TBI including type of TBI, age/sex of participants, type and length of intervention and comparator, outcome measures, and any adverse events. The risk of bias will be considered, and the strengths and weaknesses of each trial will be analyzed.

**Discussion:**

The results of this review will be considered with respect to whether there is enough evidence of benefit to merit a more definitive randomized controlled trial.

**Systematic Review Registration:** PROSPERO [CRD42022364385].

## Introduction

Traumatic brain injury (TBI) arises from a blow, jolt, or shaking of the head that disrupts normal brain function leading to physical, cognitive, and emotional effects (Centers for Disease Control and Prevention, 2015; Belanger et al., 2016; Rao et al., 2017; Public Health Agency of Canada, 2020). Globally, TBI is one of the top 10 neurological causes for high rates of disability adjusted life-years. (GBD Neurology Collaborators, 2016). In 2016, for example, there were approximately 27 million new cases of TBI globally (GBD Neurology Collaborators, 2016). Falls and road injuries were the leading causes of new cases, primarily in middle-aged and elderly, although in some countries, such as the United States and Canada, there have been rising cases of TBI in children and adolescents from sports-related injuries (Fu et al., 2016; Rao et al., 2017; Public Health Agency of Canada, 2020; Kureshi et al., 2021).

The presentation of TBI varies widely (Marshall et al., 2012). Somatic symptoms often include headache, fatigue, and signs of vestibular dysfunction, such as dizziness, vertigo, and loss of balance (Marshall et al., 2012; Xu et al., 2017; Hicks et al., 2020). Vestibular dysfunction following TBI is typically due to a peripheral cause, arising from an insult to the inner ear that disrupts the balance and head position information that is sent from the vestibulocochlear nerve to the brain. Vestibular dysfunction can also be due to a central cause when there is concomitant vertebrobasilar artery disease or a demyelintating condition (Dougherty et al., 2022). Cognitive impairment is another common complaint, resulting in decreased concentration and impaired academic and job performance (Hicks et al., 2020). When cognitive function decreases, this often results in anxiety and depression (Marshall et al., 2012).

Standard care has focused on education, physical and cognitive rest, and a gradual return to play, school, work, or usual activities (Ontario Neurotrauma Foundation, 2018; Parachute, 2021). Although it was once thought that only 20% of the TBI population had persistent symptoms at 1 year after injury (McMahon et al., 2014), the recent TRACK-TBI study in the United States found that only 47.2% of the patients with mild TBI reported full return to pre-injury levels of functioning at 1 year post-injury (Nelson et al., 2019). Even after recovery, people who have had TBI can have long-term sequelae. There is emerging evidence that TBI is associated with an increased risk of subsequent dementia and stroke (Chen et al., 2011; Mendez, 2017; Fann et al., 2018). This has led to calls for new treatments and more research.

There have been several recent advances in the treatment of TBI. Based on several systematic reviews, it is now a best practice to include early sub-threshold aerobic activity for sports-related TBI (Grool et al., 2016; Powell et al., 2020; Reid et al., 2022) and this has been applied to other causes of TBI as well (Silverberg et al., 2020). In those with persistent symptoms, there is some evidence that individually tailored multi-modal care accelerates the return to normal activities, and may include cervical and vestibular rehabilitation, cognitive rehabilitation, and supportive psychotherapy (Xu et al., 2017; Leddy et al., 2018; Silverberg et al., 2020; Reid et al., 2022).

There have also been recent advances in understanding the pathophysiology of TBI. Imaging studies have documented that 12–20% of patients with mild TBI will have macrostructural evidence of intracranial injury, such as cerebral contusions, subdural hematomas, and subarachnoid bleeds (Silverberg et al., 2020). When blood vessels are damaged, surrounding brain cells die and release damage associated molecular patterns (DAMPs) which stimulate the release of pro-inflammatory cytokines, such as IL-6 and IL-8 (Namas et al., 2015). An initial inflammatory response is part of the normal repair cycle to clear dead cells and debris. However, when there is excessive release of inflammatory cytokines, it can result in additional brain damage (Sordillo et al., 2016) and contribute to neuropsychological symptoms such as fatigue, decreased concentration, anxiety/depression, and headache (Watson, 2020).

In the United States, one in four adults with neuropsychological symptoms use mind–body practices, such as mindfulness, tai chi and yoga, and often do not discuss this with their health care provider (Purohit et al., 2013). Similar findings have been reported for other health issues in Europe (Lederer et al., 2022). Evidence is accumulating that mind-body practices can be helpful for people with TBI (Kenuk and Porter, 2017; Xu et al., 2017; Acabchuk et al., 2021; Wang et al., 2022a) and this may be due in part from reducing inflammation (Irwin and Olmstead, 2012; You et al., 2020).

Tai chi, also known as taijiquan or tai chi chuan, and its related practice qigong, are mind-body practices that have an aerobic component and a cognitive/mindfulness component (Yeh et al., 2010; Lan et al., 2013). There is an increasing body of evidence that tai chi and qigong, may help people not only with mild cognitive impairment (Zhang et al., 2018; Yang et al., 2020; Lin et al., 2021), fatigue (Wang et al., 2014; Zou et al., 2018; Zhang et al., 2019), depression and anxiety (Xiang et al., 2017), but also with other symptoms of TBI such as impaired balance (Huang and Liu, 2015; Song et al., 2015; Huang et al., 2017), and vestibular disorders (McGibbon et al., 2004; Wayne et al., 2004; Huang et al., 2019).

Studies have shown that tai chi is safe and can have health benefits for both the young and old at any fitness level (Li et al., 2001; Yeh et al., 2010; Lan et al., 2013; Zhang et al., 2018). To date, there are no systematic reviews that have focused solely on the effectiveness of tai chi/qigong for TBI. With the increased emphasis on patient-centered care, and an identified need to develop healthcare models that integrate medical and community services to support persons affected by TBI (Centers for Disease Control and Prevention, 2015), tai chi for TBI merits study. The objective of this systematic review is to identify and assess the evidence for the efficacy of tai chi and qigong for the treatment of post-traumatic brain injury.

## Methods

### Study registration

This systematic review protocol was registered on PROSPERO as Tai Chi and traumatic brain injury: A systematic review (registration number: CRD42022364385). The systematic review is designed to be in accordance with the Preferred Reporting Items for Systematic Reviews and Meta-Analyses (PRISMA) guidelines (Page et al., 2021). Amendments to the protocol will be documented in PROSPERO accordingly.

### Inclusion criteria

All clinical studies on TBI will be included that have tai chi or qigong alone or as a component of an intervention and have a comparison group, such as usual care, another exercise or non-exercise intervention, or no intervention (e.g., on a waiting list).

All participants in such studies will be included regardless of age, sex, nationality, or whether they are inpatient or outpatients. All types of tai chi or qigong and lengths of intervention will be included (Table 1).

**Table 1:** Inclusion criteria.

**Design** Randomized and non-randomized clinical trials
**Setting** Inpatient, outpatient, community-based
**Participants** Individuals diagnosed with a concussion or traumatic brain injury (mild, moderate or severe) All ages, sexes, races
**Intervention** Tai chi/qigong as the primary intervention with or without another adjuvant therapy (e.g., rehabilitation or cognitive training) All styles and durations of study
**Comparators:** Any comparison group, such as: Waiting list Standard care Any active control (such as physiotherapy, walking) Any passive control (such as health education)

### Outcome measures

As per recent trends to have patient-oriented outcomes, we have identified the preferred primary outcome as return to school, sports, work or usual activities. Secondary outcomes will include any outcome measure, such as improvement in the common symptoms of TBI, quality of life, medication usage, exacerbations, and any adverse event.

### Search strategy

We will search the following electronic databases: MEDLINE, CINAHL, Cochrane Library, Embase, China National Knowledge Infrastructure Database, Wanfang Database, Chinese Scientific Journal Database, and Chinese Biomedical Literature Database. Search terms included “Tai Chi,” “Tai Ji,” “Qigong,” “Tai Ji Quan,” “Tai Chi Chuan,” “Chi Kung,” “craniocerebral trauma,” “head or cranial trauma or injury,” “commotio cerebri,” “concussion,” “TBI,” “mild TBI,” and related terms. For example, Table 2 shows the search strategy for MEDLINE. The complete search strategy will be given in an Appendix. To identify any additional studies, the ClinicalTrials.gov will be searched for any new or planned trials, the reference lists of all studies and any systematic reviews will be examined, and studies that may be in the grey literature will be searched in OpenGrey.eu.

**Table 2:** Search strategy for database search on tai chi and traumatic brain injury.

Ovid MEDLINE	
1	exp Craniocerebral Trauma/
2	((head or crani* or capitis or brain* or forebrain* or skull* or hemisphere or intracran* or orbit* or cerebr*) adj2 (injur* or trauma* or lesion* or damage* or wound* or destruction* or oedema* or edema* or fracture* or contusio* or pressur*)).ti,ab,kf.
3	(mtbi or tbis or tbi).ti,ab,kf.
4	concuss*.ti,ab,kf.
5	commotio*.ti,ab,kf.
6	or/1–5
7	Tai Ji/ or Qigong/
8	(t’ai chi* or tai chi* or tai ji* or taiji* or qi gong* or qigong* or chi kung* or chikung*).ti,ab,kf.
9	or/7-8
10	6 and 9

### Data collection and analysis

Four authors (NL, PH, WL, and YZ) will independently screen articles against the eligibility criteria based on the title and abstract. Full-text articles will be obtained for all eligible studies and again independently assessed by all four authors against the inclusion and exclusion criteria. The authors will be blinded to each other’s decisions. Disagreements will be resolved by discussion until a consensus has been reached. If no consensus is reached, two other authors (CL and LZ) will be consulted and a final decision made. A PRISMA diagram will be created.

A standard excel data collection form will be developed for data extraction. Target population, diagnostic criteria, sample size, patient demographics, including age and sex, time from TBI to initiation of study, intervention program structure and details, control group details, all outcome measures, and follow up period will be extracted. Two authors (PH, WL) will independent extract data and two authors will independently check the extracted data (NL, CA). Study investigators will be contacted for unreported or unclear data, such as intervention and control program structure. If there is no response or the data is unavailable, the analysis will be based on the available data. Data will be reviewed, and any disagreements will be resolved by discussion among all authors. If the number of included studies is more than 10, a funnel plot will be done to assess for reporting bias.

The Cochrane risk of bias tool (RoB 2.0) will be used to assess RCTs and the risk of bias in non-randomized studies of interventions (ROBINS-I) will be used to assess observational studies (Sterne et al., 2016, 2019). The grading of recommendation, assessment, development, and evaluation (GRADE) assessment will then be conducted to evaluate the included studies. Through GRADE assessment, quality of evidence will be determined as very low, low, moderate, or high based on considerations such as study design, risk of biases, precision, consistency, directness, and other aspects reported. Any disagreements will be resolved by discussion among all authors.

An assessment of outcomes measures will be made to determine if a meta-analysis is possible. Sensitivity analyses will be conducted to assess the effect of risk of bias in the included studies, comparing studies rated at high or low risk of bias for each assessed item.

When possible, sub-groups analyses will be conducted to assess any differences in findings with respect to inpatient vs. outpatient settings, active vs. passive control groups, short term vs. long term symptoms, different types of tai chi and qigong, different age groups, and differences between men and women.

## Discussion

To our knowledge this will be the first systematic review on tai chi/qigong and traumatic brain injury. Tai chi and qigong are moderate aerobic exercises and mind-body practices that have been found to provide numerous health benefits, including improvement of many post-TBI symptoms. This systematic review will search 4 English and 4 Chinese databases to accumulate all appropriate and up-to-date research completed on this topic. Using best practice in systematic review methodology, we will use RoB 2.0 and ROBINS-I to assess the risk of bias of the studies, and GRADE to assess the certainty of the evidence evaluating the efficacy of tai chi and qigong to help people post-TBI.

In the process of finalizing this protocol, two potential limitations were identified. First, there is evidence for other conditions that tai chi is more effective when used to mitigate mild vs. moderate to severe disease. For example, tai chi is more effective decreasing the symptoms of mild cognitive impairment (Zhou et al., 2022) vs. moderate to severe dementia (Wang et al., 2022b). Similarly, tai chi may be more effective with early intervention after injury rather than late intervention. However, our pilot searches identified trials that included mild, moderate, and severe TBI patients as well as TBI patients who had had their symptoms over a wide range of time periods, from 1 month to 10 years. Given that this is the first systematic review on the topic, we decided to “cast the net wide” and not limit our review by type of TBI or time period post-TBI. However, the variations in severity and the different time periods post-TBI will be potential limitations of the literature.

The results of this study will inform whether it is warranted to conduct a larger, more definitive randomized controlled trial. The goal would be to reflect some of the recent advances in our understanding of TBI and the use of patient-based outcomes to assess the effectiveness of tai chi/qigong to mitigate the effects of TBI, one of the leading causes of non-intentional injury worldwide.

### Ethics and dissemination

In accordance with international norms, local legislation, and institutional requirements, informed consent and ethics review were not indicated as only published studies were included. All of the primary studies, however, documented informed consent and ethics approval. Dissemination plans include increasing awareness of the trial by registering the protocol and publishing both the protocol and the subsequent results and developing a dissemination strategy that includes presentations at professional venues such as conferences and a social media strategy.

## Author contributions

NL: methodology (search strategy for English databases, analysis tools), investigation, data curation, writing – original draft, and writing – review and editing. PH: conceptualization, methodology, investigation, project administration, writing – original draft, and writing – review and editing. WL: methodology (search strategy for Chinese databases, analysis tools), investigation, data curation, and writing – review and editing. CA: methodology, validation, and writing – review and editing. YZ: methodology, investigation, and writing – review and editing. LZ: methodology, validation, supervision, writing – review and editing, and funding acquisition. All authors contributed to the article and approved the final version.

## Funding

This study was partly funded by the Qi Huang Young Scholar Program (Ref. No. SCM-2020-001).

## Conflict of interest

The authors declare that this research was conducted in the absence of any commercial or financial relationships that could be construed as a potential conflict of interest.

## Publisher’s note

All claims expressed in this article are solely those of the authors and do not necessarily represent those of their affiliated organizations, or those of the publisher, the editors and the reviewers. Any product that may be evaluated in this article, or claim that may be made by its manufacturer, is not guaranteed or endorsed by the publisher.
